# AIC649 Induces a Bi-Phasic Treatment Response in the Woodchuck Model of Chronic Hepatitis B

**DOI:** 10.1371/journal.pone.0144383

**Published:** 2015-12-14

**Authors:** Daniela Paulsen, Olaf Weber, Helga Ruebsamen-Schaeff, Bud C. Tennant, Stephan Menne

**Affiliations:** 1 AiCuris GmbH & Co KG., Wuppertal, Germany; 2 Bayer Aktiengesellschaft, Leverkusen, Germany; 3 Department of Clinical Sciences, College of Veterinary Medicine, Cornell University, Ithaca, United States of America; Indiana University, UNITED STATES

## Abstract

AIC649 has been shown to directly address the antigen presenting cell arm of the host immune defense leading to a regulated cytokine release and activation of T cell responses. In the present study we analyzed the antiviral efficacy of AIC649 as well as its potential to induce functional cure in animal models for chronic hepatitis B. Hepatitis B virus transgenic mice and chronically woodchuck hepatitis virus (WHV) infected woodchucks were treated with AIC649, respectively. In the mouse system AIC649 decreased the hepatitis B virus titer as effective as the “gold standard”, Tenofovir. Interestingly, AIC649-treated chronically WHV infected woodchucks displayed a bi-phasic pattern of response: The marker for functional cure—hepatitis surface antigen—first increased but subsequently decreased even after cessation of treatment to significantly reduced levels. We hypothesize that the observed bi-phasic response pattern to AIC649 treatment reflects a physiologically “concerted”, reconstituted immune response against WHV and therefore may indicate a potential for inducing functional cure in HBV-infected patients.

## Introduction

Viruses are battled by the immune system of the infected organism in a concerted interplay of innate and adaptive responses: Antigen presenting cells (APCs) “prime” the T- and B-cell responses amongst others via cytokine release to generate an effective immune response capable of eliminating the invading virus. Functional impairment of the APC compartment results in weak and insufficient T cell responses leading to viral persistence [1] and has been recognized as a hallmark of chronic viral infections including chronic hepatitis B virus (HBV) infection [1–3]. In chronic HBV infection the high amount of soluble hepatitis B surface antigen (HBsAg) in the serum of chronic HBV patients [4] which is not suppressed by antiviral treatment is another common denominator. It has been speculated that the sheer quantity of HBsAg acting as a tolerogen might be one of the factors leading to the collapse of the functional immune response in these patients [5,6]. Consequently, decline or loss of HBsAg is used as predictor for functional cure [7–9] implemented in the international EASL HBV treatment guideline [10]. At the moment, the best anti-HBV agent to induce functional cure appears to be the immunomodulator Interferon-α (IFN-α). But even treatment with IFN-α for an extended time period of several years only results in responder rates of about 8% durable HBsAg loss at best [11]. Preclinical and clinical evidence has shown that enhancing the priming of T and natural killer (NK) cell responses by APCs might be crucial for resolution of HBV infection [1–3,12].

Parapoxvirus ovis (PPVO, Orf virus) infections or administration of inactivated PPVO (iPPVO) particle preparations have previously been found to stimulate a complex and autoregulating Th1-dominated cytokine response in mice including IFN-α, INF-γ, and tumor necrosis factor-alpha (TNF-α) thereby activating the innate arm of the immune response including APCs (i.e. dendritic cells and NK cells as well as the adaptive arm of the immune response including CD4+ and CD8+ T-cells (reviewed in [13]). This intimate interplay involving all arms of the immune system in a concerted physiological response opens up the possibility of using iPPVO as antiviral treatment against unrelated chronic viral infections. It has already been shown that the iPPVO driven responses translate into antiviral efficacy against unrelated viruses e.g., herpes viruses [14–16], hepatitis C virus [17], and HBV [15,17]. In the present study we analyzed the potency of AIC649, an iPPVO particle preparation, to reduce the viral titer in HBV transgenic (tg) mice in comparison to the “gold standard” in HBV treatment Tenofovir. However, the HBV tg mouse model is an artificial model to determine antiviral efficacy but does not allow the analysis of immune control over HBV since the HBV transgene is “inbuilt” and therefore regarded as “self” by the organism. One of the experimental animal model systems to examine immune-mediated functional cure of HBV is the woodchuck chronically infected with WHV. Therefore, we also present the efficacy of AIC649 in chronic WHV carrier woodchucks.

## Materials and Methods

### Mice studies

#### Animals

BALB/c AnN mice (Charles River) were used to characterize the kinetics of cytokine release. Mice transgenic for the human HBV (HBV tg mice) carrying a frameshift mutation (GC) at position 2916/2917, designation: [Tg (HBV1.3 fsX-3’5’)] [18], (bred in-house) were used to characterize the correlation of cytokine release with HBV titer reduction. HBV tg mice were housed in individually ventilated cages (Tecniplast, Germany) and handled under a laminar flow for additional protection in a Biosafety Level 2 facility. Food, water, and bedding were sterilized before being provided to animals. Mice were handled according to federal Guidelines and under the approval of the Bezirksregierung Recklinghausen / Duesseldorf. For anesthesia of the mice and sampling see the respective section below.

#### Test Materials

iPPVO strain NZ-2 (AIC649) lot 02V19 or lot 0010310, respectively, were reconstituted with 1.1 ml water for injection (equaling a dose of 2.5 x 10^6^ U/ml (measured by ELISA) or 5 x 10^8^ viral particles / ml (measured by qEM), respectively. ELISA titer differs from qEM determined titer by a factor of ~100). Administration volume i.p.: 200 μl, administration volume i.m.: 50 μl. Tenofovir tablets (Viread^®^, 245 mg, Gilead) were ground and dissolved in 2% DMSO / 98% Tylose (0.5% Methylcellulose / 99.5% PBS). As negative control the Tenofovir formulation buffer 2% DMSO / 98% Tylose (0.5% Methylcellulose / 99.5% PBS) was used. Dilutions were performed using 0.9% NaCl solution (pyrogen-free, Fresenius Kabi).

#### Anesthesia and Sampling

Mice were anesthetized for retrobulbar blood sampling using an Isoflurane Vaporizer. For collection of terminal heart puncture blood or organs, mice were anaesthetized with isoflurane and subsequently sacrificed by CO_2_ exposure. Retrobulbar (100–150 μl) and heart puncture (400–500 μl) blood samples were collected into a Microvette 300 LH or Microvette 500 LH, respectively, followed by separation of plasma via centrifugation (10 min, 2000g, 4°C). Liver tissue was taken and snap frozen in liquid N_2_. All samples were stored at -80°C until further use.

#### HBV DNA Extraction

Viral DNA was extracted from 50 μl plasma or 25 mg liver and eluted in 50 μl AE buffer (plasma) using the DNeasy 96 Blood & Tissue Kit or 320 μl AE buffer (liver) using the DNeasy Tissue Kit (both kits: Qiagen) according to the manufacturer’s instructions. The eluates were stored at -20°C until further use.

#### HBV qPCR

Eluted viral DNA was subjected to quantitative polymerase chain reaction (qPCR) using the LightCycler 480 Probes Master PCR kit (Roche) according to the manufacturer’s instructions to determine the HBV copy number. HBV specific primers (TIB MOLBIOL) used included: Forward primer “ayw-F”: 5’-CTG TAC CAA ACC TTC GGA CGG-3’; reverse primer “ayw-R“: 5’-AGG AGA AAC GGG CTG AGG C-3’; FAM labeled probe “ayw-T-BBQ”: FAM-CCA TCA TCC TGG GCT TTC GGA AAA TT-BBQ. One PCR reaction sample with a total volume of 20 μl contained 5 μl DNA eluate and 15 μl master mix (comprising 0.3μM of the forward primer, 0.3μM of the reverse primer, 0.15μM of the FAM labeled probe). qPCR was carried out on the Roche LightCycler^®^480 using the following protocol: Pre-incubation for 1 min at 95°C, amplification: (10 sec at 95°C, 50 sec at 60°C, 1 sec at 70°C) x 45 cycles, cooling for 10 sec at 40°C. Standard curves were generated using HBV plasmid pcH-9/3091 [19]. All samples were tested in duplicate. The detection limit of the assay is ~50 HBV DNA copies (using standards ranging from 250–2.5 x 10^7^ copy numbers). Results are expressed as HBV DNA copies / 10μl plasma or HBV DNA copies / 100ng total liver DNA (normalized to negative control).

#### Cytokines

Plasma samples were analyzed using the cytometric bead arrays for mouse Th1/Th2 cytokines or inflammation (Becton Dickinson), respectively, according to the manufacturer’s instructions. Concentrations of TNF-α or IFN-γ are given in pg/ml plasma.

### Woodchuck studies

#### Animals

The woodchucks used for this study were born to WHV-negative females and reared in laboratory animal facilities at Cornell University. Woodchucks used in the single dose study with AIC649 were adult animals, of both gender, and naïve for WHV (WHV-negative). For the efficacy study, neonatal woodchucks were subcutaneously infected at 3 days of age with the WHV7P1 inoculum containing 5 x 10^6^ WID_50_ of WHV strain 7 [20] and were then raised to adulthood (approximately 1 year of age). Woodchucks were considered chronic WHV carriers based on the persistent presence of WHsAg, WHV DNA, antibody to WHV core antigen (anti-WHc) in serum, the absence of antibody to WHV surface antigen (anti-WHs). WHV carrier status was confirmed prior to initiation of treatment. Ten chronic WHV carrier woodchucks with approximately equal numbers of males and females were stratified on the basis of gender, body weight, and viral load into two experimental groups of five animals each. All woodchucks were free of hepatocellular carcinoma (HCC) at the beginning of the study based on hepatic ultrasound examination and normal serum activity of gamma-glutamyl-transferase (GGT). The animal protocol and all procedures involving woodchucks were approved by the Cornell University IACUC and adhered to the national guidelines of the Animal Welfare Act, the Guide for the Care and Use of Laboratory, and the American Veterinary Medical Association. Prior to euthanasia, woodchucks were anesthetized by intramuscular injection of ketamine (50 mg/kg) and xylazine (5 mg/kg). Thereafter, woodchucks were euthanized by an overdose of pentobarbitol administered by intracardiac injection. This method is consistent with the recommendations of the Panel on Euthanasia of the American Veterinary Medical Association.

#### Test Materials

AIC649 (Lot No. 01V18) was manufactured and provided by Bayer HealthCare AG. Following reconstitution in pyrogen-free water for injection, 1 ml of AIC649 (equaling a dose of 3.9 x 10^6^ U/ml (measured by ELISA)) was administered by intramuscular (i.m) injection twice weekly to each of the 5 woodchucks within the treatment group. Vehicle (Lot No. 01V19) also was reconstituted in pyrogen-free water and 1 ml of vehicle was administered i.m. twice weekly to each of the 5 woodchucks within the control group.

#### Blood Collection/Anesthesia

Blood samples were obtained under general anesthesia (ketamine 50 mg/kg and xylazine 5 mg/kg i.m.) at two weeks and one week prior to treatment, on the first day of AIC649 or vehicle administration (“day 0”), and then weekly during the 8-week treatment period. Thereafter, samples were obtained weekly until week 12, and then biweekly until the end of the study at week 24. Serum biochemical measurements included GGT, alkaline phosphatase (ALP), alanine aminotransferase (ALT), aspartate aminotransferase (AST), and sorbitol dehydrogenase (SDH). Serum activities of AST, ALT, and SDH are markers of hepatocellular injury in woodchucks [21].

#### WHV Serum Viremia Levels

WHV DNA was quantified by dot blot hybridization using three replicate 10 μl volumes of undiluted serum compared with a standard dilution series of WHV recombinant DNA plasmid, pWHV8 [20] (assay sensitivity, ≥ 1.0 x 10^7^ WHV genome equivalents per ml [WHVge/ml]) as described previously [22].

#### WHV Serology

WHsAg, anti-WHc, and anti-WHs in serum were determined using WHV-specific enzyme immunoassays [23]. Dilutions of serum were used to ensure detection of all markers under saturating conditions.

#### T cell proliferation

Peripheral blood mononuclear cells (PBMCs) were isolated from whole blood by Ficoll-paque density gradient centrifugation prior, during, and following treatment with AIC649 or vehicle. Cells were cultured and stimulated for 4 days with recombinant WHcAg (rWHcAg; 1.0 μg/ml), native 22-nm WHsAg (2.0 μg/ml), and selected synthetic peptides (WHc peptide C100-119 and WHs peptide S226-245; each 10.0 μg/ml) as described recently [24,25]. Dividing PBMCs were then labelled overnight with [2-^3^H]adenine (Amersham Pharmacia Biotech, Inc., Arlington Heights, IL). Results for stimulated cultures were expressed as a stimulation index, which was determined by dividing the total counts per minute in the presence of stimulator by that in the absence of stimulator. Results were further represented as a fold change relative to pre-treatment (“day 0").

#### Cytokine Induction

Whole blood samples were obtained prior to, during, and following treatment. During the treatment period, blood was collected 13–14 hours or at indicated time points after administration of AIC649 or vehicle. Total RNA was isolated using the QIAamp RNA Blood Mini Kit (Qiagen, Valencia, CA) according to the manufacturer’s instructions. Following DNase I treatment (Invitrogen, San Diego, CA) and reverse transcription with the TaqMan Reverse Transcription Reagents kit (Applied Biosystems, Foster City, CA) using oligo-d(T)16, cDNA was amplified by real-time PCR using 2X Core Reagent kit (Applied Biosystems) and the ABI PRISM 7000 Sequence Detection instrument (Applied Biosystems) as described previously [26]. Woodchuck-specific primer and probes for the detection of interleukin-2 (IL-2), IFN-γ, and TNF-α were used [26]. Expression of 18S rRNA (Applied Biosystems) was used to normalize target gene expression. Transcript levels of target genes were determined by the formula 2^*ΔCT*^, where ΔC_T_ indicates the difference in the threshold cycle between 18S rRNA and target gene expression. Cytokine transcript levels were then calculated as fold change relative to pre-treatment (“day 0”).

### Statistical Analysis

The mean levels of WHV DNA, WHsAg, liver enzymes, T cell proliferation, and cytokine transcripts at each sampling date were compared between the treatment and control groups with Student's *t* test (two-tailed). *P* values of < 0.05 were considered statistically significant.

## Results

**Fig 1:**
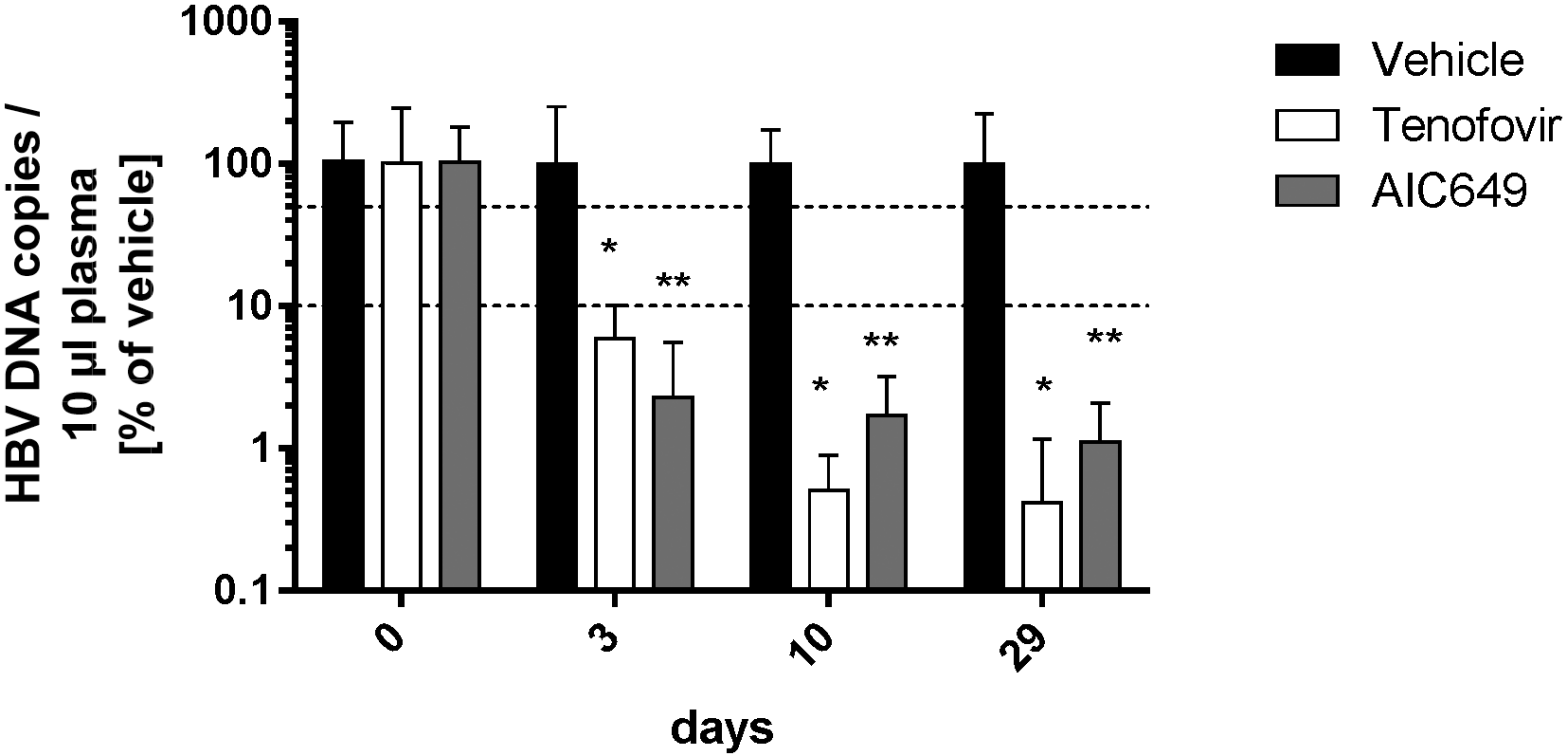
AIC649 reduces the HBV titer in HBV tg mice as effective as Tenofovir. HBV tg mice were treated i.p. with AIC649 (1 x 10^8^ viral particles / dose) twice weekly for 9 times in total. Tenofovir was administered twice daily by oral gavage at a total concentration of 100 mg/day. Negative control: Tenofovir vehicle, administration analogous to Tenofovir. Mice were sacrificed at the indicated time points and plasma samples were subjected to HBV DNA determination. HBV titers are expressed in % of the respective negative vehicle group +/- SD. * p < 0.05, ** p < 0.01 (vs respective treatment on day 0).

Inactivated parapoxvirus ovis preparations (iPPVO), including AIC649, have previously been described to reduce the HBV titer in the HBV tg mouse model which is clearly attributable to the induction of antivirally active cytokines, e.g. IFN-γ and TNF-α, by iPPVO [15,17]. AIC649 has just recently been shown to be more potent in reducing the HBV titer in HBV tg mice than other iPPVO strains [17]. Here we analyzed the capacity of the immunomodulator AIC649 to reduce the HBV titer in comparison to highly potent direct acting antivirals (DAAs): We tested AIC649 against the “gold standard” Tenofovir. HBV tg mice were treated either twice weekly with AIC649 or twice daily with Tenofovir or vehicle for a period of 29 days. Both treatment regimens resulted in a significant reduction of the HBV titer compared to the control (Fig 1). Twice weekly treatment with AIC649 had an antiviral effect similar to that of twice daily treatment with Tenofovir. Similar results have been obtained comparing AIC649 with Entecavir (data not shown).

**Fig 2:**
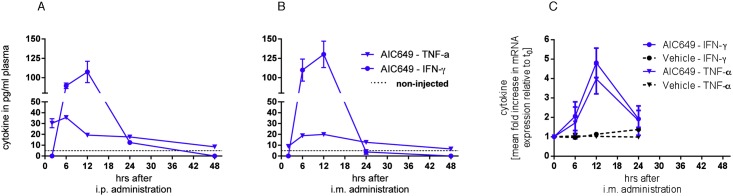
AIC649 induces similar cytokine release kinetics in mice and woodchucks. Three healthy BALB/c mice / time point were treated once with 1 x 10^5^ U AIC649 i.p. (A) or 2.5 x 10^4^ U AIC649 i.m. (B). Mice were sacrificed at the indicated time points and plasma samples were pooled. The pooled plasma samples were subjected to duplicate cytokine determination. Plasma samples from non-injected mice were used as controls. Given are the means +/- SD. (C): Three healthy woodchucks were treated once with 3.9 x 10^6^ U AIC649 /ml or vehicle. Blood was drawn at the indicated time points and subjected to determination of cytokine transcript levels. Results are given as mean fold change relative to pre-treatment (t = 0) levels +/- SD.

As the HBV tg mouse model is only valid to analyze the antiviral efficacy of a compound, we wanted to use the chronically WHV-infected woodchuck model to analyze the efficacy of AIC649 to induce immune-mediated functional cure. First we needed to analyze if AIC649 would be active in woodchucks at all. As cytokine induction can be used as a marker for AIC649 activity we conducted experiments in healthy mice and healthy woodchucks to bridge between species as well as between routes of administrations. In mice, AIC649 induced the same pattern of cytokine release regardless of the route of administration: i.p. and i.m. treatment with AIC649 resulted in IFN-γ and TNF-α release peaking after 6 (TNF-α) to 12 hrs (IFN-γ) after administration (Fig 2A and 2B). AIC649 also led to cytokine release in healthy woodchucks: i.m. treatment resulted in IFN-γ and TNF-α release peaking 12 hrs after administration (Fig 2C). Having confirmed AIC649 activity in woodchucks we were confident to test AIC649 administered i.m. in chronically WHV-infected woodchucks.

**Fig 3:**
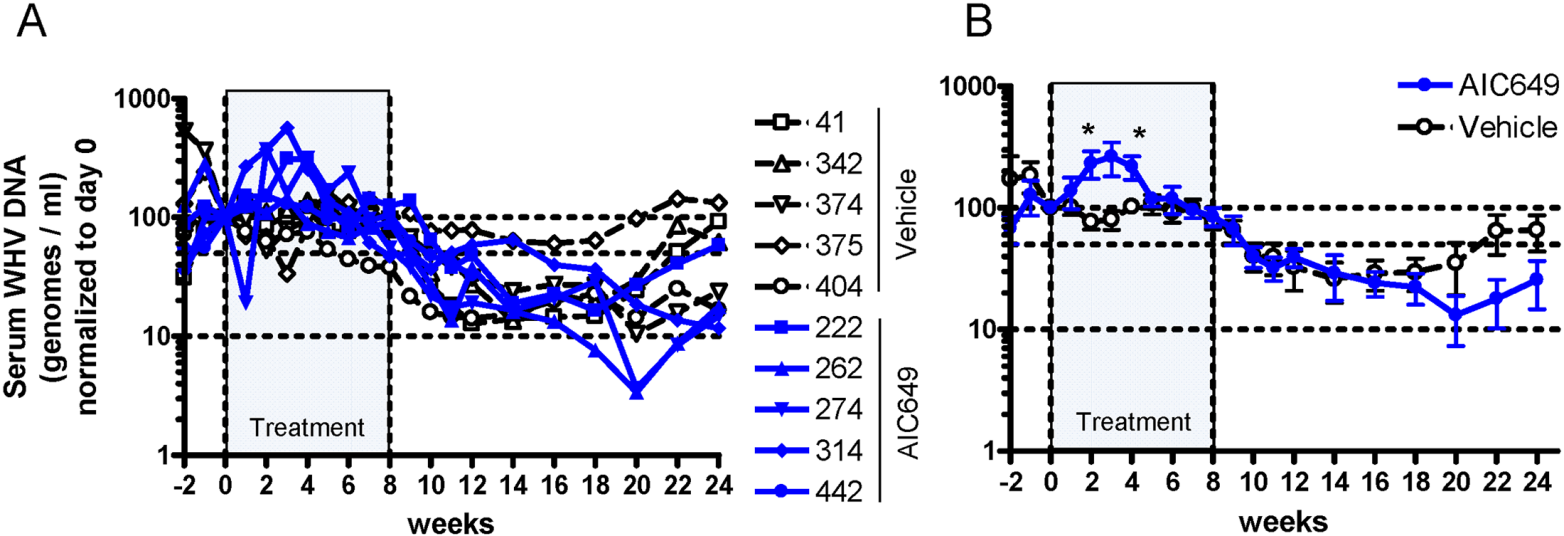
AIC649 induces a bi-phasic treatment effect on viremia levels in chronic WHV carrier woodchucks. AIC649 (3.9 x 10^6^ Units (ELISA-based titer)) or vehicle were administered i.m. twice weekly for 8 weeks (marked “Treatment”). At the indicated time points woodchucks were bled and serum was subjected to determination of WHV viral load. Values were normalized to individual baseline at day 0 and are given in %. n = 5 / group. Dotted lines: 100%, 50%, or 10% level. (A) Serum WHV DNA concentrations of individual woodchucks (identified by numbers) in the vehicle and AIC649 groups. (B) Group means of WHV DNA concentration +/- standard error of the mean (SEM). *p < 0.05.

The antiviral activity of AIC649 was assessed against WHV in chronically infected woodchucks during an 8-week period of twice weekly, i.m. treatment. Woodchucks were followed thereafter for an additional 16 weeks until the end of the 24 week period of study. When compared to vehicle-treated control woodchucks treatment with AIC649 did not result in significant reductions of serum WHV DNA, neither during the treatment period nor for most of the follow-up period (Fig 3A and 3B). To the contrary, WHV DNA levels in individual AIC649-treated woodchucks increased following the initiation of treatment, and the increase in the mean was statistically significant at weeks 2 and 4 compared to the control group (*p* < 0.05). However, towards the end of the follow-up period, viremia levels in AIC649-treated woodchucks markedly declined between weeks 16 and 20, and stayed at lower levels than those in control woodchucks until the end of the study.

**Fig 4:**
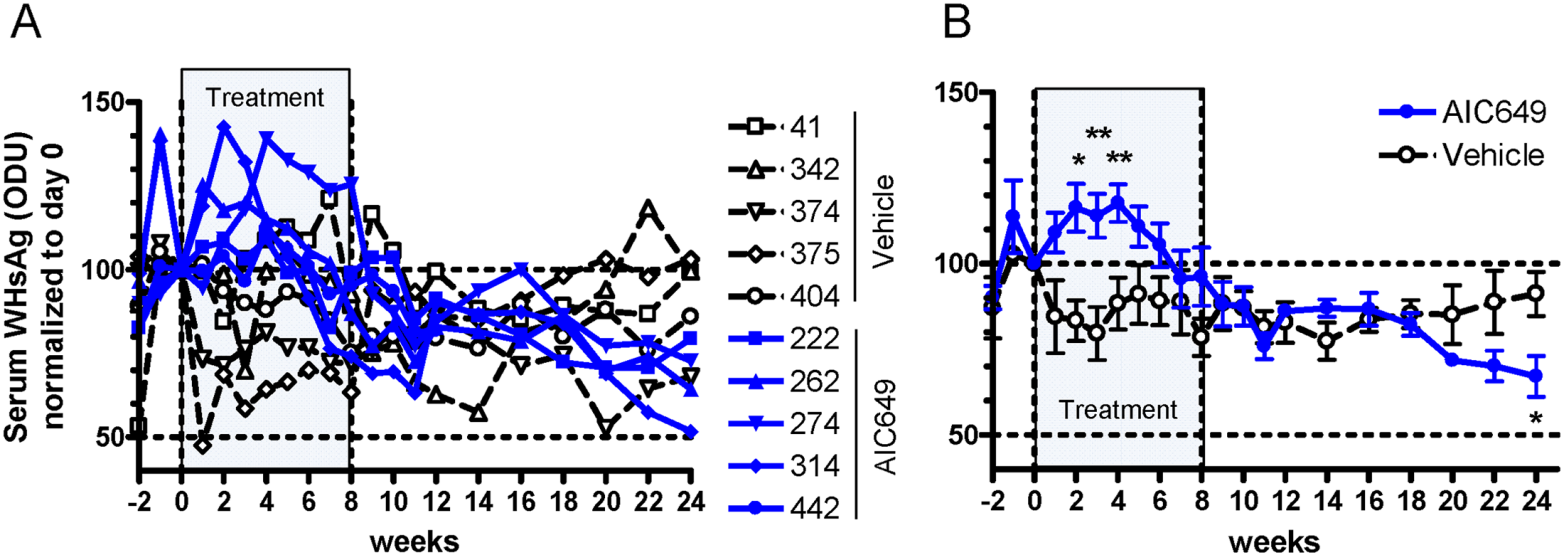
AIC649 induces a bi-phasic treatment effect on antigenemia levels in chronic WHV carrier woodchucks. AIC649 (3.9 x 10^6^ Units (ELISA-based titer)) or vehicle were administered i.m. twice weekly for 8 weeks (marked “Treatment”). At the indicated time points woodchucks were bled and serum was subjected to determination of WHsAg load. Values were normalized to individual baseline at day 0 and are given in %. n = 5 / group. Dotted lines: 100% or 50% level. (A) Serum WHsAg concentrations of individual woodchucks (identified by numbers) in the vehicle and AIC649 groups. (B) Group means of WHsAg concentration +/- SEM. *p < 0.05, **p < 0.01. ODU, optical density units.

A similar pattern of an initial increase and then decrease in viral markers was also observed for serum WHsAg in woodchucks of the treatment group (Fig 4A and 4B). Compared to vehicle-treated woodchucks, antigenemia levels in individual AIC649-treated woodchucks increased between weeks 2 and 4 of treatment, and the difference in the means between both groups was again statistically significant (*p* < 0.01 to *p* < 0.05). Thereafter, antigenemia levels declined in individual woodchucks of the treatment group and segregated from those of the control group starting as early as week 18. At the end of the study at week 24, the decline in mean WHsAg level of the treatment group was significantly different (*p* < 0.05) from the control group.

Seroconversion to antibodies against WHsAg (anti-WHs) was not detected in any woodchuck of either treated or control group (data not shown). Interestingly, the preexisting antibody response to WHcAg (anti-WHc) in chronic WHV carrier woodchucks transiently increased in AIC649-treated animals around the same time at which the transient increases in serum viral markers were observed (data not shown).

**Fig 5:**
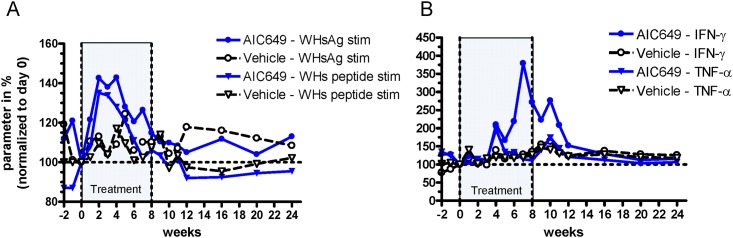
AIC649 treatment induces cell-mediated immune responses in chronic WHV carrier woodchucks. AIC649 (3.9 x 10^6^ Units (ELISA-based titer)) or vehicle were administered i.m. twice weekly for 8 weeks (marked “Treatment”). At the indicated time points woodchucks were bled and PBMCs were subjected to determination of T cell proliferation after stimulation, whereas whole blood was used to analyze transcript levels of cytokines. Values were normalized to individual baseline at day 0 and are given in %. n = 5 / group. Dotted lines: 100% level. (A) Percent-change in mean T cell proliferation level from baseline at day 0 for woodchucks in the vehicle and AIC649 groups, respectively, following stimulation of PBMCs with WHsAg (WHsAg stim) or WHs peptide S226-245 (WHs peptide stim). (B) Percent-change in mean IFN-γ and TNF-α transcript level from baseline at day 0 in blood of woodchucks from both groups.

Furthermore, AIC649 administration induced cell-mediated immune responses in chronic WHV carrier woodchucks as shown by the increases in T cell proliferation and IFN-γ expression in PBMCs during and following treatment (Fig 5A and 5B). Comparable changes were not observed in the control group. Stimulation of PBMCs with the entire surface antigen (WHsAg) or a WHs-derived peptide containing a woodchuck T cell epitope [27] resulted in transiently increased T cell proliferation between weeks 1 and 6 that coincided with the increases in WHV DNA and WHsAg during AIC649 treatment. PBMC stimulation with the entire core antigen (WHcAg) and a selected T cell epitope-bearing core-derived peptide revealed the same pattern of T cell proliferation as observed with WHsAg and the WHs peptide (data not shown). Transiently increased IFN-γ transcripts were observed in AIC649-treated woodchucks between weeks 4 and 12 indicating that an antiviral cytokine response was induced by AIC649 and that this response was sustained beyond the end of treatment. This was in contrast to TNF-α transcripts that did not appear consistently up-regulated by AIC649 compared to controls except for a transient and minor increase in the AIC649 treated group at week 4.

**Fig 6:**
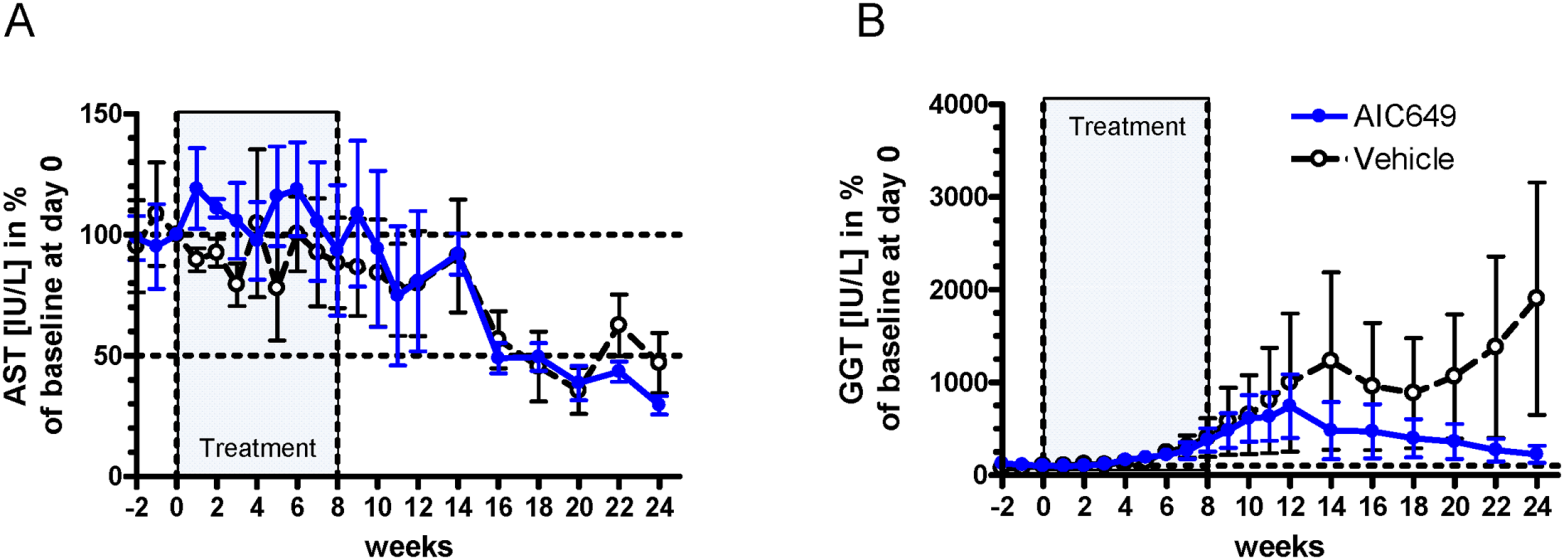
AIC649 treatment is associated with changes in serum activities of liver enzymes in chronic WHV carrier woodchucks. AIC649 (3.9 x 10^6^ Units (ELISA-based titer)) or vehicle were administered i.m. twice weekly for 8 weeks (marked “Treatment”). At the indicated time points woodchucks were bled and serum was used to analyze levels of liver enzymes. Values were normalized to individual baseline at day 0 and are given in %. n = 5 / group. Dotted lines: 100% or 50% level. (A). Group means of AST concentration +/- SEM. (B) Group means of GGT concentration +/- SEM.

Associated with the treatment-induced transient increases in serum viral markers and cell-mediated immune responses, serum activity of the liver enzyme AST was also transiently elevated in AIC649-treated woodchucks suggesting increased liver injury, presumably associated with immune mediated viral clearance. Following the end of treatment, AST activity declined and became normalized in treated woodchucks with the mean serum level comparable to that of control woodchucks (Fig 6A). Comparable changes were also observed for the serum activities of SDH and ALT (data not shown). ALP levels in AIC649-treated woodchucks did not remarkably change while those in control woodchucks were elevated through most of the study (data not shown). In addition, serum activity of GGT, an established oncofetal marker of liver tumor development in woodchucks with chronic WHV infection, increased in a few woodchucks of both groups, especially during the end of AIC649 or vehicle treatment and the early follow-up periods (week 6–12) (Fig 6B). One AIC649-treated woodchuck was found dead after week 12 of the study and the cause of death of this animal was attributed to the development of HCC. However, while the mean serum activity of GGT continued to increase in control animals it declined in AIC649-treated woodchucks.

## Discussion

AIC649 is an inactivated parapoxvirus ovis particle preparation with distinct immunological activities; i.e. induction of IFN-α, INF-γ, and TNF-α release, activation of immune cells including dendritic cells, NK cells, CD4+, and CD8+ T-cells (reviewed in [13]). Albeit TLR-9 has been described as being the “sensing” receptor for iPPVO [28] the virus mediates the stimulation of the immune system by addressing TLR-dependent and -independent pathways [29]. The activation of the immune system by iPPVO in general and AIC649 in particular translates into antiviral efficacy in the HBV tg mouse model [15,17]. This observation is in line with previously published data: (1) Infection of HBV tg mice with adenovirus, murine cytomegalovirus, or lymphocytic choriomeningitis virus [30,31] as well as (2) direct administration of cytokines [32–35] or immunomodulatory substances including the TLR-9 ligand CpG [34,36] reduces the HBV titer. Of note, the immunomodulator AIC649 given only twice weekly is as efficacious in the HBV tg mouse model as twice daily administration of the DAAs Tenofovir (Fig 1) or Entecavir (data not shown) which are currently regarded as gold standards in the treatment of chronic hepatitis B in humans.

The HBV tg mouse model is used for determination of general antiviral efficacy. Treatment of mice leads to reduction of HBV titer but not to reduction of HBsAg (loss of HBsAg is the main goal of antiviral therapy and is regarded as a predictor for functional cure [10]). This is mirroring the limitations of this transgene model where HBV is regarded as “self” and immune control per definition cannot lead to reduction of HBsAg except if tolerance is broken [37] and autoimmune mechanisms are triggered. Therefore, the HBV tg model does not mimic the immunological situation of patients with chronic hepatitis B who display a dysregulated immune response to HBV [1]. Neonatal woodchucks following vertical infection with WHV undergo immunological changes that are similar to those of humans. In chronically infected woodchucks, persistent WHV replication, constitutive WHsAg expression, and impaired NK cell and/or T cell responses all are observed [38]. To test AIC649’s ability to trigger functional cure in an immune tolerant setting, we wanted to test this immunomodulator in the woodchuck model for chronic hepatitis B. In a pilot experiment we were able to show that treatment of healthy woodchucks with AIC649 did lead to cytokine release (Fig 2C) which is a prerequisite for analyzing AIC649 efficacy in this species. In addition, we observed that i.m. administration of AIC649 in woodchucks displayed the same kinetic of cytokine release regardless of the route of administration (i.p. or i.m.) in mice (Fig 2A–2C). Therefore, we were confident to test the efficacy of AIC649 to induce functional cure in chronically WHV-infected woodchucks.

Treatment of chronic WHV infected woodchucks with AIC649 surprisingly revealed a bi-phasic pattern of response: The WHV DNA as well as WHsAg first increased and peaked about 2–4 weeks after the start of treatment. Only subsequently both parameters started to decline and in fact continued to decline even further after cessation of therapy at week 8 and during most (WHV DNA, Fig 3) or the entire period of follow-up until the end of the study at week 24 (WHsAg Fig 4). Although WHsAg level were reduced at the end of the study seroconversion to antibodies against WHsAg (anti-WHs) was not detected.

The initial increase in WHV DNA and WHsAg was paralleled by transiently increased T cell proliferation between weeks 1 and 6 and subsequent by a transiently increased cytokine response, suggesting active cell mediated immunity associated with immune-mediated clearance of WHV-infected hepatocytes immediately after the start of treatment (Fig 5). This explanation is supported by the serum activity of the liver enzymes AST (Fig 6A), SDH and ALT (data not shown) which also were transiently elevated in AIC649-treated woodchucks suggesting increased liver injury. On the other hand, the immune mediated clearance did not lead to persistent liver injury or HCC development: To the contrary, we found the mean liver enzyme GGT declining in AIC649-treated animals, especially during the follow-up period which contrasted with the increasing mean serum activities of GGT in the control animals (Fig 6B). These data fit to the observed amelioration or prevention of liver fibrosis in non-virally induced rat models of liver fibrosis by treatment with AIC649 or iPPVO strain D1701 [17,39].

We are aware that the observed effects might deem not strong enough: WHsAg reduction reaches statistical significance only at the end of the observation period whereas WHV even fails to reach statistical significance from vehicle at the end of the experiment. The development of anti-WHs antibodies could not be detected. An explanation could be that the study was terminated too early and at a time before WHsAg reduction was complete as WHsAg loss precedes development of anti-WHs antibodies. Another explanation could be that the dose of AIC649 used for treatment was not high enough to lead to the necessary level of WHsAg reduction. A follow-up study with a longer observation period and higher doses of AIC649 is warranted to clarify the final outcome.

Nevertheless, our interpretation is that the summary of all parameters—viral as well as immunological—obtained in our study and resulting in the observed bi-phasic AIC649 response pattern most likely reflects the natural course of the physiologically staggered anti-WHV immune response. Initiation of the cell mediated immune response is complemented by cytokine production leading to release / increase of WHV-DNA and WHsAg from destroyed infected cells. This is correlating with transient increases in certain liver enzymes (AST, ALT, SDH). Destruction of infected cells then ultimately led to the observed decrease of WHV DNA and WHsAg. In addition, (1) the pattern is quite uniform in all animals treated and (2) the decrease of WHV DNA and WHsAg continues after cessation of AIC649 therapy. The observation of a bi-phasic pattern is in line with observations from the clinics as chronically HBV infected patients resolving HBV experience so called “flares”: increases in liver enzymes and / or increases in HBV DNA preceding reconstitution of anti-HBV immune response [40,41]. To our knowledge this pattern of bi-phasic response which continued after cessation of treatment has not been shown for any of the marketed anti-HBV drugs or drugs in development—neither DAAs nor immunomodulators [42,43]. In addition—and again to the best of our knowledge—other immunomodulators as e.g. GS-9620 (a TLR-7 agonist under development by Gilead) display antiviral efficacy but might be hampered by tolerability issues [42]. AIC649, however, displays an interesting treatment response pattern combined with very good tolerability. Consequently, AiCuris has initiated the clinical development for AIC649 in chronic hepatitis B patients.
